# Cloning and Characterization of the Human *USP22* Gene Promoter

**DOI:** 10.1371/journal.pone.0052716

**Published:** 2012-12-26

**Authors:** Jianjun Xiong, Xiangxin Che, Xueqin Li, Huan Yu, Zhen Gong, Weidong Li

**Affiliations:** 1 College of Basic Medical Science, Jiujiang University, Jiujiang, China; 2 Key Laboratory of Jiangxi for the Systems Bio-medicine, Jiujiang, China; Niels Bohr Institute, Denmark

## Abstract

Ubiquitin-specific processing enzyme 22 (USP22) plays a direct role in regulating cell cycle, and its overexpression has been reported to be involved in tumor progression. However, little is known about the regulation of *USP22* transcription. In this study, we cloned and characterized the human *USP22* promoter. Using 5′ RACE (rapid amplification of cDNA ends) analysis, the transcriptional initiation site was identified. Promoter deletion analysis showed that the sequence between −210 and −7 contains the basal promoter for *USP22* in human fibroblast and tumor cells. Surprisingly, mutations in a putative Sp1 binding site immediately upstream of the *USP22* transcriptional start site (−13 to −7) resulted in a significant induction of promoter activity. Further study revealed that Sp1 binds to this site in human normal fibroblast cells, and treatment with the Sp1 inhibitor mithramycin A led to a marked increase in *USP22* transcript levels. Forced expression of exogenous Sp1 repressed the USP22 promoter activity in HeLa cells. In contrast, knockdown of Sp1 enhanced USP22 promoter activity and mRNA levels. These data suggest that Sp1 is a crucial regulator of *USP22* transcription.

## Introduction

Ubiquitin-specific processing enzyme 22 (*USP22*), also named *USP3L* or *KIAA1063*) is a novel deubiquitinating enzyme, which cleaves ubiquitin (Ub) from Ub-conjugated protein substrates [1]. In eukaryotes, *USP22* functions as an enzymatic subunit of the hSAGA transcriptional cofactor complex [2]. It has been documented that a key regulator of cell cycle, *p21*, is regulated by *USP22* [3], and depletion of *USP22* results in a G1 phase cell-cycle arrest in human tumor cells [2]. Furthermore, *USP22* participation in regulating shelterin protein turnover and telomere maintenance has also been demonstrated [4].

In humans, the *USP22* gene is located on chromosome 17 and consists of 14 exons [1]. Northern blot analyses demonstrated that *USP22* was expressed moderately in various tissues, including heart, skeletal muscle, and weakly in lung and liver [1]. Mitogen activation or virus infection in normal T and B lymphocytes may stimulate *USP22* expression to promote cell immortalization [5], suggesting that the regulation of *USP22* gene expression occurs mainly at the transcriptional level. More importantly, *USP22* is considered as one of the putative cancer stem cell markers [2], and changes in the expression of *USP22* have been linked with metastatic potential and therapeutic outcome in human cancer [6], [7]. However, the mechanisms leading to USP22 transcriptional activation, particularly in human tumor cells, are still unknown.

In this article, we tried to analyze *USP22* gene regulation at the transcriptional level. By generating a number of 5′- and 3′-deletion constructs to delineate the promoter region, we identified a 203-bp fragment necessary for basal transcriptional activity. In addition, we provided evidence for the first time that Sp1 is involved in the regulation of human *USP22* expression.

## Materials and Methods

### Cell cultures

Normal human lung fibroblast (HFL1) cells and the human cervical cancer cell line (HeLa) were from the American Tissue Culture Collection (ATCC) and were cultured in Dulbecco's modified Eagle's medium (DMEM, Invitrogen) supplemented with 10% fetal bovine serum (GIBCO) at 37°C in a 5% CO_2_ and 95% air in an incubator.

### 5′ Rapid amplification of cDNA ends (5′-RACE)

A 5′-RACE system was performed according to the manufacturer's instructions (TaKaRa Bio). Briefly, 2 µg of total RNA extracted from HeLa cells was treated with calf intestinal phosphatase to remove the free 5′-phosphate group. Tobacco acid pyrophosphatase was then used to specifically remove the cap structure from the full-length mRNA, leaving a 5′-monophosphate. A RNA oligonucleotide adaptor was next ligated to the newly decapped mRNA by T4 RNA ligase. With the ligated RNA as a template, *USP22* cDNA was synthesized by reverse transcription using M-MLV reverse transcriptase and random primers. The resulting cDNA was then amplified by nested PCR using LA Taq DNA polymerase as well as the human *USP22* gene primer (reverse) and the adaptor primer (forward) provided by the manufacturer. The gene-specific antisense inner primer 5′-CCGCAGGTTCTGCTTCCAGTTGT-3′ (+117 to +95) and outer primer 5′-CTCCTCCTTGGCGATTATTT-3′ (+393 to +374) were complementary to the *USP22* cDNA sequence. The PCR products were analyzed on agarose gels and cloned into the pMD-18T vector (TaKaRa) for sequencing to determine the transcriptional start site(s).

### Genome DNA isolation and cloning of the human *USP22* promoter

Genome DNA was isolated from the HeLa cells using a DNA extraction kit (QIAGEN) and dissolved in water. Based upon our findings regarding the location of the transcriptional start site, a 2880-bp fragment from −2828 to +52 in the 5′-flanking region of the *USP22* gene was generated by PCR. The amplified DNA was cloned into a pMD 18-T simple vector and verified by direct sequencing. Other deletion fragments were generated by PCR using this plasmid DNA as a template (see Table 1 for PCR primer sequences). The PCR products were gel-purified, digested with KpnI and BglII, and subcloned into the pGL3-basic firefly luciferase vector (Promega). All the sequences of the cloned promoter region were confirmed by DNA sequencing. The sequence of the *USP22* promoter was analyzed for the presence of consensus transcription factor binding sites using the MatInspector program (Genomatix Software GmbH).

**Table 1:** Primers used in the generation of promoter luciferase constructs.

Construct	Primer	Sequence 5′→3′
p-2880/+52	sense-2880	AGGTACCGATAGGGTTTCATCACATTG
p-2880/−1306	antisense-1306	GGAGATCTTGTGGCCAAGACAAATTGCC
p-1306/+52	sense-1306	AAAGCTTTATCCCAGTCGTCAGTCC
p-866/+52	sense-866	CGGTACCTTTGACTTTATTGGGTTGAG
p-866/−326	antisense-326	GGAGATCTGGCTCTCAGTATAGTCCGTC
p-595/+52	sense-595	AGGTACCCTGCAAACAGCTCCCGATTA
p-326/+52	sense-326	CGGTACCTATGACAATAGCCGAAGGTG
p-210/+52	sense-210	AGGTACCGTCTACCCAGAGCCTAACGG
p-7/+52	sense-7	AGGTACCTGCCTGCCTTGCAGCCTCCC
common	antisense+52	GGAGATCTGCGGAGGCCGGACAAAGATGGG

Site-directed mutagenesis to inactivate the Sp1-binding sites at positions −13 to −7 of the promoter were carried out within the p-210/+52 construct according to the MutanBEST Kit methodology (TaKaRa Bio) with the following primers: mutant Sp1 site forward, 5′-GATCGGTGCCTGCCTTGCA-3′; deletion Sp1 site forward, 5′-TGCCTGCCTTGCAGCCTCCC-3′; Sp1 reverse, 5′-CCGAGCTGCGGCTGCTGCGGA-3′. All mutations were confirmed by DNA sequencing.

### Transfections and dual luciferase reporter assay

HFL1 cells and HeLa cells were plated in 24-well plates 24 hours before transfection with 0.5 µg of various *USP22* promoter constructs and 0.1 µg of pRL-TK (Promega) using Lipofectamine 2000 (Invitrogen) in each well. All transfection experiments were repeated five times. Twenty-four hours after transfection, cells were washed in phosphate-buffered saline and lysed for 30 min at room temperature using passive lysis buffer (Promega). Luciferase activity was determined using the dual luciferase reporter assay system (Promega). Normalized luciferase activity was expressed as the ratio of firefly luciferase activity to *Renilla* luciferase for each sample.

### Chromatin Immunoprecipitation (ChIP) Assay

ChIP assays were performed according to the EZ-ChIP Kit (Millipore) manufacturer instructions. Briefly, HFL1 cells were fixed by adding formaldehyde to a final concentration of 1% and incubated by modest shaking for 30 min at room temperature. Thereafter, cells were washed twice with cold phosphate-buffered saline. The pellet was resuspended and lysed, and nuclei were isolated and sonicated until the chromatin had an average length of 500–1500 bp. After centrifugation, the supernatant was incubated with 3 µg of antibody against Sp1 overnight at 4°C for immunoprecipitation. The following day, magnetic protein-G beads were added and the mix was further incubated at 4°C for 1 hour. After appropriate washing, the antibody-transcription factor-DNA complex was eluted from the beads, formaldehyde cross-links were reversed, and proteins were digested with proteinase K at 67°C overnight. DNA was purified and used for PCR with primers 5′ GTCTACCCAGAGCCTAACGG 3′ and 5′ GCGGAGGCCGGACAAAGATGGG 3′.

### Construction of Sp1 expression plasmids

Total RNA was obtained from human HeLa cells and reverse-transcribed with M-MLV Reverse Transcriptase primed by oligo (dT)_15_. Primers used in the subsequent PCR amplification of Sp1 cDNA were: forward, 5- GAAGCTTATGAGCGACCAAGATCACTCCATG-3, and reverse, 5-GGAATTCTCAGAAGCCATTGCCACTGA-3. The PCR products were digested with HindIII and EcoRI and inserted into pCDNA3.1(+).

A truncated form of Sp1 (ΔSp1) lacking its DNA-binding domain and consisting of three zinc-fingers (amino acids 621–708) [8] was obtained by PCR-mediated deletion by the following sequence: 5′-CAGAATAAGAAGGGAGGCCCA-3′ and 5′-AGGATCCCCCGAGCCCCTTCC-3′. All constructs were confirmed by DNA sequencing.

### RNA interference and real-time PCR

One day before transfection, HFL1 cells were plated at a density of 5×10^4^ cells per well in 24-well plates, then transfected with 20 nM of Control siRNA (sc-37007, Santa Cruz Biotechnology, Inc.) or human SP1-specific siRNA (sc-29487, Santa Cruz Biotechnology, Inc.) using the siRNA Reagent System (sc-45064, Santa Cruz Biotechnology, Inc.) according to the manufacturer's instructions. After 24 hours, the medium was changed and the cells were further transfected with 0.2 µg of a p-210/+52 construct using lipofectamine 2000 and incubated for another 24 h. Luciferase assays were performed following the manufacturer's instructions. In parallel, wells were harvested after siRNA transfection. Total RNA was isolated from cells using TRIzol Reagent (Invitrogen), and reverse-transcribed as above. Real-time PCR was performed using SYBR Green PCR Master Mix (Takara Bio) on an ABI 7500 Real-Time PCR System (Applied Biosystems). USP22 primer pairs were as follows: forward, 5′ - GTGTCTTCTTCGGCTGTTTA -3′, reverse, 5′-CTCCTCCTTGGCGATTATTT-3′. Sp1 primer pairs were: forward, 5′-GCCGCTCCCAACTTACAGAA-3′; reverse, 5′ -TGCCTCCACTTCCTCGATTT- 3′

### Statistical analysis

Data are presented as the mean ± SEM. Statistical differences between sample means were determined using unpaired, two-tailed Student's *t* test. Significance was set at a probability value less than 0.05.

## Results

### Identification of the 5′-flanking region of the human *USP22* gene

**Figure 1:**
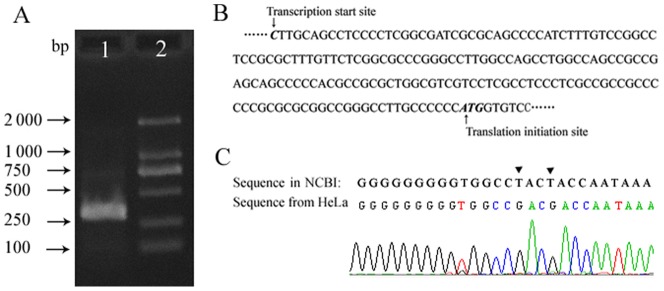
Identification of the transcriptional start site of the *USP22* gene. **A.** Results of 5′ RACE experiments on total RNA from HeLa cells; DNA sequences were detected by gel electrophoresis using 2% agarose. Lane 1. A single DNA band of 260 bp was detected; Lane 2. DL2000 marker. **B.** Sequence of *USP22* gene depicting the positions of the transcriptional start sites. **C.** Compared with the existing information in the human genome database, the DNA sequence of the HeLa clone contains two mutations, A–C and A–C, at positions 280 and 283 upstream from the transcriptional start site.

The transcription initiation site(s) for *USP22* have not yet been confirmed. We performed 5′- RACE to amplify the 5′-end of the *USP22* cDNA from the HeLa cell mRNA. After reverse transcription and nested PCR, a band of approximately 260 bp was obtained and then cloned into a pMD18-T vector. The sequencing results from 25 randomly selected clones revealed that a single transcriptional start site was located at 176 bp upstream of ATG in mature *USP22* mRNA (Fig. 1A), which is identical to the *USP22* genomic sequence (Fig. 1B). Sequence analysis showed that the region upstream of the transcriptional start site includes one GC box but lacks typical TATA boxes.

Based upon our findings regarding the transcriptional start site, we amplified by PCR from genomic DNA from HeLa cells a 2880-bp DNA fragment containing the region of the transcriptional start site, and inserted it into pMD-18T vectors for DNA sequencing. Surprisingly, we found two novel single-nucleotide polymorphisms (SNP) in the clone that are not documented in the existing human genome sequence in NCBI. The sequence from HeLa cells contained two mutations; T-G and T-G, at positions 283 and 280 upstream (positions −283 and −280, respectively) of the transcriptional start site (Fig. 1C). Genomic DNA from an additional five healthy Chinese were isolated to detect these SNPs. Results showed that the two SNPs were present in all of the genomic DNAs.

**Figure 2:**
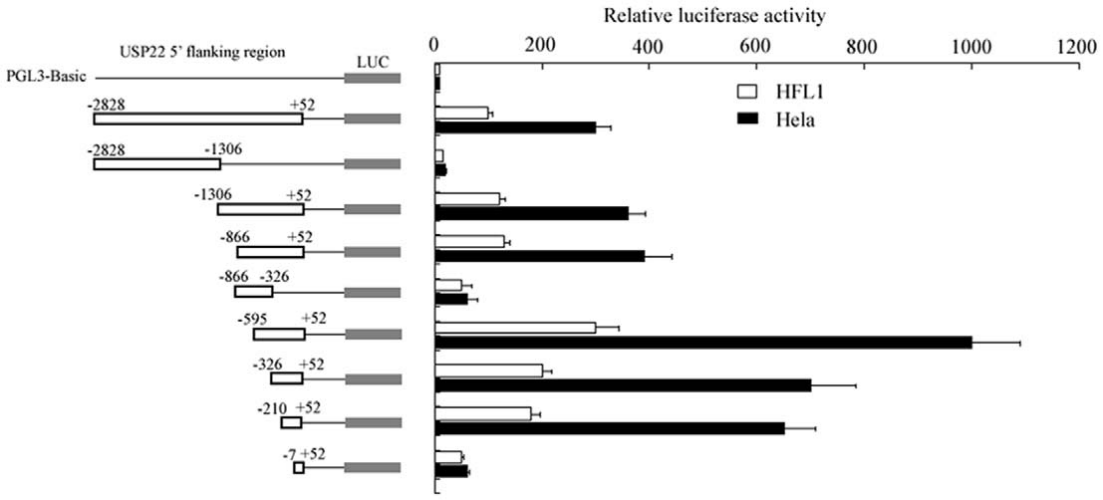
Luciferase reporter assay for the *USP22* gene promoter. A series of fragments of the 5′-flanking USP22 promoter region is schematized. Each promoter-reporter construct or the promoter-less plasmid pGL3-basic, was co-transfected with pRL-TK into HFL1 and HeLa cells. Luciferase activities were measured after 24 h and normalized for transfection efficiency. The luciferase activity of each construct is presented relative to the pGL3-basic activity.

To analyze the promoter activity of the 5′-flanking region of USP22, various truncated versions of the *USP22* promoter were amplified and cloned into the luciferase reporter vector pGL-3 Basic. These constructs were transfected into human lung fibroblast (HFL1) or human tumor cells (HeLa) to determine the sequence elements required for *USP22* promoter activity. As shown in Fig. 2, the longest construct, p-2828/+52, was able to drive expression of the luciferase reporter gene in HFL1 cells one hundred times higher than the pGL3-Basic construct, which indicates the transcriptional functionality of the promoter. In human tumor cells (HeLa), transfection with this construct also resulted in significantly higher activity (300 times greater than for pGL3-Basic). The 3′-truncated construct p-2828/+1306, lacking the 3′-end 1522 bp from P-2828/+52, displayed dramatically inhibited promoter activity in both HFL1 and HeLa cells, indicating that the region −1306 to +52 was essential in driving the *USP22* promoter. To further characterize the p-1306/+52 region, sequential 5′-deletion constructs from p-1306 were investigated. In HFL1 cells, the construct p-595/+52 showed the highest activity among the *USP22* promoter constructs, which was approximately 300 times higher than the activity shown by pGL3-Basic. The longer constructs, p-866/+52 and p-1306/+52, showed only 20% of the activity shown by p-595/+52, suggesting that the core promoter is present in the −595 to +52 regions, and that the sequence between −596 and −866 might contain a negative element(s) inhibiting promoter activity. The luciferase activity of p-326/+52 and p-210/+52 was approximately two-fold greater than that of p-2828/+52, but 30% lower than that of p-595/+52. The shortest construct, P-7/+52, demonstrated no luciferase activity. These data indicate that the promoter region between −210 and −7 is required for basal transcriptional activity of the USP22 gene, but further analysis is required.

In HeLa cells, the construct p-595/+52 showed approximately 900 times higher activity than pGL3-Basic, which was also the highest activity among all of the constructs. Similarly, the construct p-210/+52 was the shortest fragment exhibiting a high promoter activity, showing 700 times higher activity than pGL3-Basic.

### Mutations in the Sp1-binding site of the *USP22* promoter lead to elevated transcription

**Figure 3:**
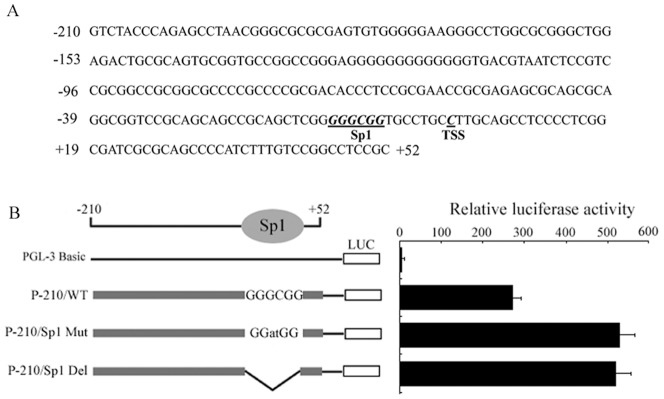
Mutation analyses of the Sp1-binding site. **A.** Nucleotide sequence and structural organization of the *USP22* gene core promoter region. Putative binding sites for the transcriptional factors are underlined. **B.** Luciferase activity expressed by the Sp1 site-directed mutant and deletion mutants relative to pGL3-basic activity.

Since the region −210 to −7 is required for basal transcriptional activity of the *USP22* gene, we analyzed the putative transcriptional factor binding sites in this region using MatInspector software. As shown in Fig. 3A, this region contains a Sp1-binding site, a CREB/ATF binding site, and a c-Myb binding site. Among these motifs, the Sp1-binding site attracted our attention because this site and the transcriptional start site are juxtaposed, and Sp1 has been reported to activate promoters lacking a TATA box. To evaluate the significance of the Sp1-binding site for *USP22* transcription, we substituted GATCGG for the consensus sequence GGGCGG or deleted this consensus sequence in the parental vector p-210/+52 to generate p-210/Sp1mut or p-210/Sp1del constructs, respectively. After transient transfection, the p-210/Sp1mut and p-210/Sp1del constructs showed an approximately 170% higher luciferase activity in HFL1 cells compared with the p-210/+52 construct, suggesting that this DNA motif plays a negative role toward *US*P22 promoter activity (Fig. 3 B).

### Interaction between Sp1 and the *USP22* Promoter

**Figure 4:**
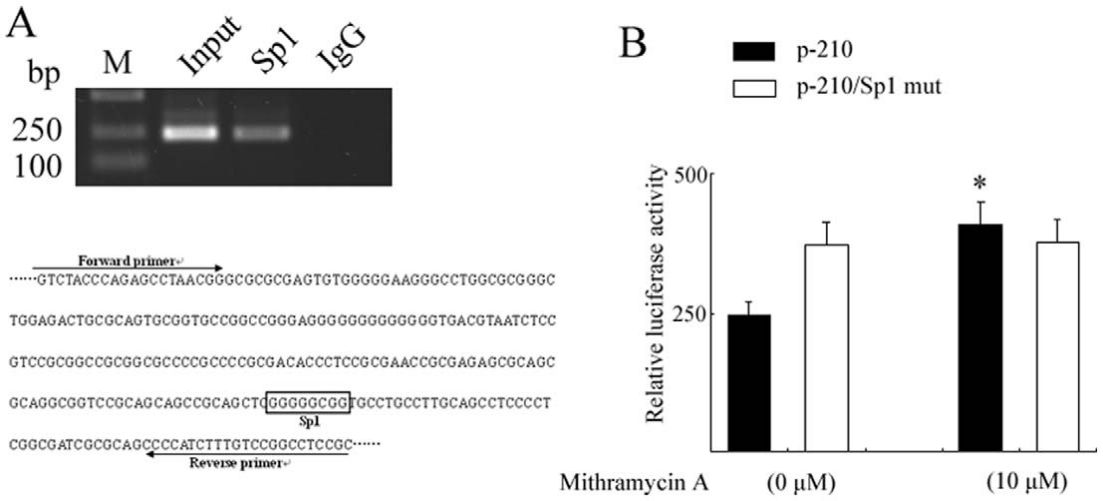
Determination of Sp1 binding to the *USP22* gene promoter. **A.** DNA was isolated from HFL1 cells in each group and immunoprecipitated with antibodies against Sp1, RNA polymerase II or nonspecific rat IgG. Input and immunoprecipitated DNAs were then PCR amplified using primer pairs covering the Sp1-binding site. **B.** HFL1 cells expressing p-210 or p-210/Sp1mut constructs were treated with 10 um of mithramycin A or vehicle. Luciferase activity was determined 24 h later (*, *p*<0.05 *vs.* vehicle treatment).

Transcription factor Sp1 has a high affinity for the GC box; we therefore determined whether cellular Sp1 binds to this DNA motif in the *USP22* promoter region. A ChIP assay was carried out using an antibody against human Sp1. Immunoprecipitation of cross-linked chromatin from HFL1 cells with an anti-Sp1 antibody followed by PCR amplification of the region (the sequence between-210 and +52) confirmed that the endogenous Sp1 protein does bind to this region of the *USP22* promoter in HFL1 (Fig. 4A).

Furthermore, we used the cell-permeable agent mithramycin A to inhibit the binding of Sp1 to DNA, since mithramycin A binds to GC-rich DNA sequences, thereby precluding the DNA binding of Sp1 [9]. After HFL1 cells were transfected with a p-210/+52 plasmid and treated with 10 µM mithramycin A for 12 hours, the luciferase activity was significantly higher compared with that in vehicle-treated cells (Fig. 4B).

### Effect of Sp1 on *USP22* promoter activity

**Figure 5:**
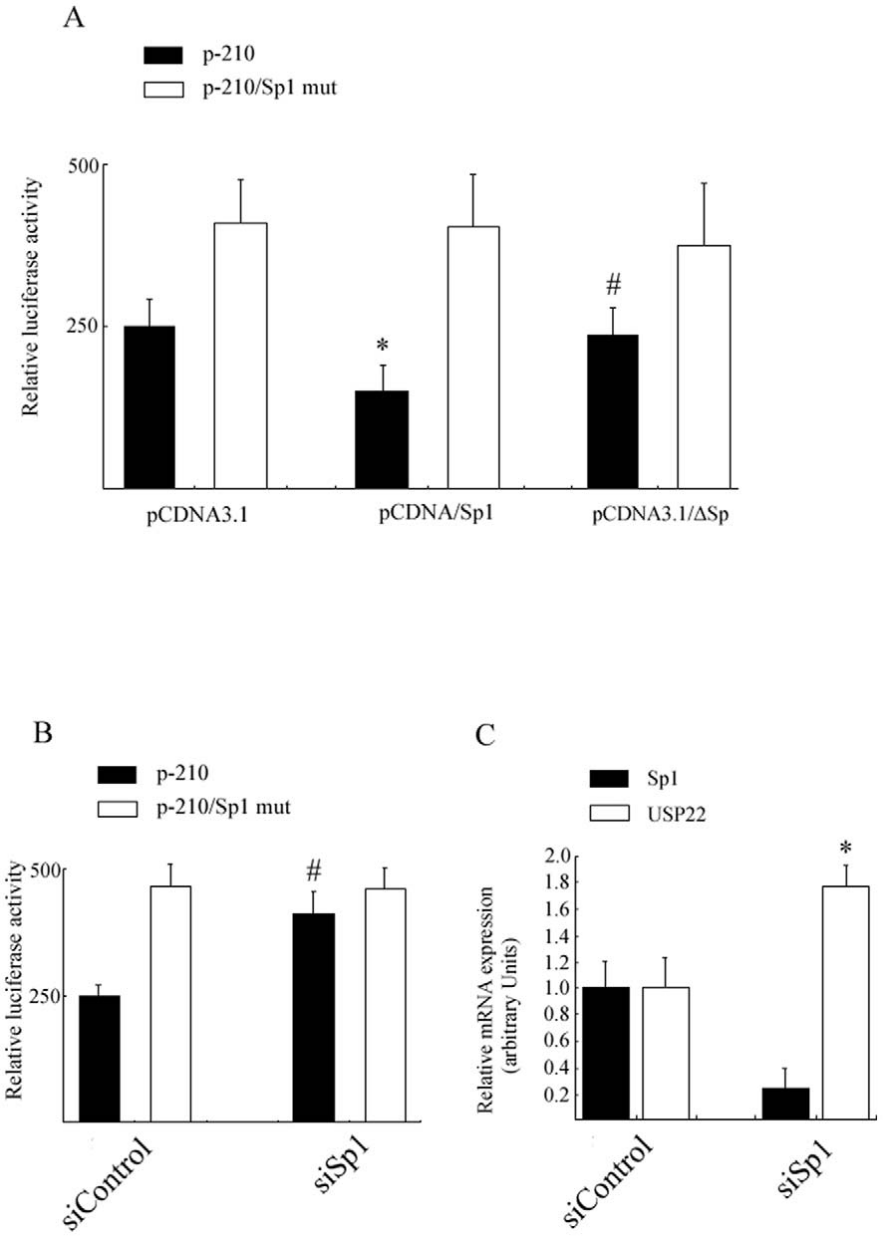
Effects of Sp1 on the *USP22* promoter. **A.** HeLa cells were co-transfected with *USP22* promoter plasmids p-210 or p-210/Sp1 mut and expression plasmids for either Sp1, ΔSp or an empty vector (pCDNA3.1). Luciferase activity was determined 24 h later. Luciferase activities are expressed as the percentage of p-210 relative to pCDNA3.1 activity. (*, *p*<0.05 *vs.* pCDNA3.1; #, *p*>0.05 *vs.* pCDNA3.1). **B.** HFL1 cells were transfected with Sp1 siRNA or non-targeting control siRNA. *Sp1* and *USP22* mRNA expression was determined by real-time PCR (*, *p*<0.05 *vs.* siControl). **C.** HFL1 cells were co-transfected with p-210 or p-210/sp1 mut and Sp1 siRNA or non-targeting control siRNA, and with pRL-RK. Luciferase activities are expressed as the percentage of p-210 activity in the presence of control siRNA (#, *p*<0.05 *vs.* siControl).

Since HeLa cells showed high *USP22* expression, we examined the effect of Sp1 on *USP22* transcriptional regulation by co-transfecting HeLa cells with the p-210/+52 construct and a CMV- driven Sp1 expression vector. Forced expression of exogenous Sp1 repressed *USP22* promoter activity by 40%. The decline in *USP22* promoter activity was not observed in the p-210/Sp1mut construct lacking the Sp1 site. Additionally, we constructed a truncated form of Sp1 (ΔSp1) lacking its DNA-binding domain and which consists of three zinc-fingers [10]. HeLa cells were transfected with either full-length Sp1, ΔSp1, or mock vector and examined 24 hours later. As expected, ΔSp1 transfection did not affect the *USP22* promoter activity (Fig. 5A).

To investigate whether reduction of Sp1 protein can enhance *USP22* promoter activity, we down-regulated Sp1 expression by RNA interference (RNAi). HFL1 cells were co-transfected for 24 hours with siRNA that specifically targeted Sp1 and P-210/+52. As shown in Fig. 5B, we achieved about a 70% reduction in Sp1 protein, and the luciferase activity was significantly increased in siRNA-transfected HFL1 cells; however no change in detectable luciferase activity was observed in the control siRNA-transfected cells. To determine the effect of Sp1 on endogenous *USP22* expression, the mRNA levels of *USP22* were detected by real-time PCR after siRNA transfection. As shown in Fig. 5C, the siRNA-mediated knockdown of Sp1 led to a highly significant induction of *USP22* mRNA compared with controls (over 1.8 fold).

## Discussion

In this study, we identified and characterized the human *USP22* promoter and its activity. For the first step, a single transcriptional initiation site of the human *USP22* gene was confirmed. The sequence between the transcriptional initiation site and translational start site is identical to the *USP22* DNA sequence, suggesting that this region is a major transcriptional start site of the human *USP22* gene. Because the *USP22* gene is abnormally activated in various human tumor cells [7], its promoter activity was investigated in both cultured HFL1 and HeLa cell lines. Using a luciferase reporter system, we found that the promoter constructs of *USP22* displayed relatively higher activity in HeLa cells than in HFL1, cells indicating abnormal transcriptional activity of the *USP22* gene in human tumor cells. By generating a series of 5′ deletions, we confirmed that the region between −210 and −7 is required for the basal activity of the *USP22* promoter both in HFL1 and HeLa cells; we therefore focused our attention on this region. Bioinformatics analysis shows that, within this region, there are putative binding sites for transcription factors Sp1, CREB/ATF, and Myb. Among these, a single Sp1-binding site located at −7 to −13 of the *USP22* promoter drew our attention. Unexpectedly, mutation or deletion of this motif resulted in a significant enhancement in *USP22* promoter activity, suggesting that the Sp1-binding site functions as a negative element for *USP22* gene transcription.

The transcription factor Sp1 binds to various promoters with high affinity, and acts as an activator or an inhibitor depending upon the genes [11], [12], [13]. Accumulating evidence shows that Sp1 plays a role in tumorigenesis. For example, Sp1 activity is increased following phosphorylation by oncogenic signaling pathways such as RAS/RAF/MAPK and PI3K/Akt [14], [15], [16]. In addition, Sp1 is overexpressed or hyperactivated in a number of human tumors [17], [18]. Multiple genes involved in tumorigenesis, such as those in cell growth, apoptosis and angiogenesis, are found to be promoted by Sp1, including survivin [19], VEGF [15] and NME5 [20]. Based on these findings, we initially hypothesized that Sp1 might play a positive role in *USP22* gene transcription, since *USP22* is up-regulated in most human tumor cells. However, the experimental results do not support our hypothesis. The binding of Sp1 to the DNA motif juxtaposed with the transcriptional start site (which consequently prevents transcriptional initiation), might be a mechanism for Sp1's negative role in *USP22* transcription. Another possibility is the formation of secondary structures caused by the high GC content, leading to a reduced transcriptional level.

To verify these assumptions, we performed ChIP assays. Results showed that Sp1 binds to the *USP22* promoter in fibroblasts, suggesting that Sp1-DNA interaction may be required for *USP22* repression. We next performed several experiments on different aspects to further verify this hypothesis. First, we inhibited Sp1 binding activity with mithramycin A. When HFL1 cells expressing the construct p-210/+52 were treated with mithramycin A for 12 h, luciferase activity was significantly increased compared with that of vehicle-treated cells. Second, forced expression of Sp1 decreased p-210/+52 luciferase activity; however, the truncated Sp1 lacking its DNA-binding domain did not affect luciferase activity. Third, the knock-down of Sp1 expression induced *USP22* promoter activity. Finally, we confirmed that over-expression of Sp1 inhibited the endogenous *USP22* expression in human tumor cells. Collectively, these results strongly indicate that Sp1 plays a negative role and that its DNA-binding activity is a key step needed for *USP22* transcription.

Although Sp1 has been described primarily as a transcriptional activator, recent results show that Sp1 also plays a role in transcriptional repression in multiple promoters [13], [21], [22]. Sp1 represses gene expression via protein-protein interaction, or interplay with other transcription factors such as Rb [13] or p53 [23]. However, we did not identify any partner protein that cooperates with Sp1 to repress *USP22* expression; this remains to be elucidated in future work. Conversely, Sp1 binding to DNA is reversible and adjustable. Growing evidence indicates that the posttranslational modifications of Sp1 protein (such as phosphorylation [24], acetylation [25], sumoylation [26], and glycosylation [27]), can influence its DNA-binding ability and gene transactivation. Understanding whether or how these posttranslational modifications influence Sp1-DNA interaction is crucial for uncovering *USP22* transcription mechanisms.

For some time Sp1 protein expression was believed to be a critical factor in tumor development, growth and metastasis, but other studies argue that its over-expression is detrimental to various cells. This controversy indicates an intricate role for Sp1 in cellular physiology. How Sp1 induces cell apoptosis is poorly understood currently. Multiple mechanisms are probably involved in Sp1-induced apoptosis. For example, the *Bcl-x* gene (which encodes an anti-apoptotic protein), was suppressed in Sp1-overexpressing Baf-3 cells [8]. P53 was also found to accumulate in Sp1- overexpressing cancer cells [28]. In addition to these proposed mechanisms, our studies indicate that the increased Sp1 expression may, via binding to the promoter, contribute to the inhibition of USP22 expression, thus inducing cell-cycle arrest.

In conclusion, we have cloned and characterized the proximal human *USP22* promoter. Our data demonstrate for the first time that the transcription factor Sp1 is involved in the negative regulation of human *USP22* expression. These findings provide new insights into Sp1's function and regulation of *USP22* transcription

## References

[1] Lee HJ, Kim MS, Shin JM, Park TJ, Chung HM, et al (2006) The expression patterns of deubiquitinating enzymes, USP22 and Usp22. Gene Expr Patterns 6: 277–284. 16378762 10.1016/j.modgep.2005.07.007

[2] Zhang XY, Varthi M, Sykes SM, Phillips C, Warzecha C, et al (2008) The putative cancer stem cell marker USP22 is a subunit of the human SAGA complex required for activated transcription and cell-cycle progression. Mol Cell 29: 102–111. 18206973 10.1016/j.molcel.2007.12.015 PMC2254522

[3] Atanassov BS, Dent SY (2011) USP22 regulates cell proliferation by deubiquitinating the transcriptional regulator FBP1. EMBO Rep 12: 924–930. 21779003 10.1038/embor.2011.140 PMC3166460

[4] Atanassov BS, Evrard YA, Multani AS, Zhang Z, Tora L, et al (2009) Gcn5 and SAGA regulate shelterin protein turnover and telomere maintenance. Mol Cell 35: 352–364. 19683498 10.1016/j.molcel.2009.06.015 PMC2749492

[5] Ovaa H, Kessler BM, Rolen U, Galardy PJ, Ploegh HL, et al (2004) Activity-based ubiquitin-specific protease (USP) profiling of virus-infected and malignant human cells. Proc Natl Acad Sci U S A 101: 2253–2258. 14982996 10.1073/pnas.0308411100 PMC356937

[6] Glinsky GV (2005) Death-from-cancer signatures and stem cell contribution to metastatic cancer. Cell Cycle 4: 1171–1175. 16082216 10.4161/cc.4.9.2001

[7] Glinsky GV (2006) Genomic models of metastatic cancer: functional analysis of death-from-cancer signature genes reveals aneuploid, anoikis-resistant, metastasis-enabling phenotype with altered cell cycle control and activated Polycomb Group (PcG) protein chromatin silencing pathway. Cell Cycle 5: 1208–1216. 16760651 10.4161/cc.5.11.2796

[8] Deniaud E, Baguet J, Mathieu AL, Pages G, Marvel J, et al (2006) Overexpression of Sp1 transcription factor induces apoptosis. Oncogene 25: 7096–7105. 16715126 10.1038/sj.onc.1209696

[9] Miller DM, Polansky DA, Thomas SD, Ray R, Campbell VW, et al (1987) Mithramycin selectively inhibits transcription of G-C containing DNA. Am J Med Sci 294: 388–394. 2962490 10.1097/00000441-198711000-00015

[10] Philipsen S, Suske G (1999) A tale of three fingers: the family of mammalian Sp/XKLF transcription factors. Nucleic Acids Res 27: 2991–3000. 10454592 10.1093/nar/27.15.2991 PMC148522

[11] Kim S, Kang JK, Kim YK, Seo DW, Ahn SH, et al (2006) Histone deacetylase inhibitor apicidin induces cyclin E expression through Sp1 sites. Biochem Biophys Res Commun 342: 1168–1173. 16516150 10.1016/j.bbrc.2006.02.081

[12] Banchio C, Schang LM, Vance DE (2003) Activation of CTP:phosphocholine cytidylyltransferase alpha expression during the S phase of the cell cycle is mediated by the transcription factor Sp1. J Biol Chem 278: 32457–32464. 12794070 10.1074/jbc.M304810200

[13] Law AY, Yeung BH, Ching LY, Wong CK (2011) Sp1 is a transcription repressor to stanniocalcin-1 expression in TSA-treated human colon cancer cells, HT29. J Cell Biochem 112: 2089–2096. 21465530 10.1002/jcb.23127

[14] Milanini-Mongiat J, Pouyssegur J, Pages G (2002) Identification of two Sp1 phosphorylation sites for p42/p44 mitogen-activated protein kinases: their implication in vascular endothelial growth factor gene transcription. J Biol Chem 277: 20631–20639. 11904305 10.1074/jbc.M201753200

[15] Pore N, Liu S, Shu HK, Li B, Haas-Kogan D, et al (2004) Sp1 is involved in Akt-mediated induction of VEGF expression through an HIF-1-independent mechanism. Mol Biol Cell 15: 4841–4853. 15342781 10.1091/mbc.E04-05-0374 PMC524732

[16] Chuang CW, Pan MR, Hou MF, Hung WC (2012) Cyclooxygenase-2 up-regulates CCR7 expression via AKT-mediated phosphorylation and activation of Sp1 in breast cancer cells. J Cell Physiol 228 2: 341–348. 10.1002/jcp.24136 22718198

[17] Hsu TI, Wang MC, Chen SY, Yeh YM, Su WC, et al (2011) Sp1 expression regulates lung tumor progression. Oncogene 568: 1–16. 10.1038/onc.2011.568 PMC3432230 22158040

[18] Wang L, Wei D, Huang S, Peng Z, Le X, et al (2003) Transcription factor Sp1 expression is a significant predictor of survival in human gastric cancer. Clin Cancer Res 9: 6371–6380. 14695137

[19] Liu YL, Jiang SX, Yang YM, Xu H, Liu JL, et al (2012) USP22 Acts as an Oncogene by the Activation of BMI-1-Mediated INK4a/ARF Pathway and Akt Pathway. Cell Biochem Biophys 62: 229–235. 21928107 10.1007/s12013-011-9287-0

[20] Li F, Jiang Z, Wang K, Guo J, Hu G, et al (2012) Transactivation of the human NME5 gene by Sp1 in pancreatic cancer cells. Gene 503: 200–207. 22564704 10.1016/j.gene.2012.04.088

[21] Mottet D, Pirotte S, Lamour V, Hagedorn M, Javerzat S, et al (2009) HDAC4 represses p21(WAF1/Cip1) expression in human cancer cells through a Sp1-dependent, p53-independent mechanism. Oncogene 28: 243–256. 18850004 10.1038/onc.2008.371

[22] Cole LK, Vance DE (2010) A role for Sp1 in transcriptional regulation of phosphatidylethanolamine N-methyltransferase in liver and 3T3-L1 adipocytes. J Biol Chem 285: 11880–11891. 20150657 10.1074/jbc.M110.109843 PMC2852925

[23] Dalvai M, Mondesert O, Bourdon JC, Ducommun B, Dozier C (2011) Cdc25B is negatively regulated by p53 through Sp1 and NF-Y transcription factors. Oncogene 30: 2282–2288. 21242964 10.1038/onc.2010.588

[24] Guo L, Eviatar-Ribak T, Miskimins R (2010) Sp1 phosphorylation is involved in myelin basic protein gene transcription. J Neurosci Res 88: 3233–3242. 20882567 10.1002/jnr.22486

[25] Waby JS, Chirakkal H, Yu C, Griffiths GJ, Benson RS, et al (2010) Sp1 acetylation is associated with loss of DNA binding at promoters associated with cell cycle arrest and cell death in a colon cell line. Mol Cancer 9: 275. 20950428 10.1186/1476-4598-9-275 PMC2972244

[26] Spengler ML, Brattain MG (2006) Sumoylation inhibits cleavage of Sp1 N-terminal negative regulatory domain and inhibits Sp1-dependent transcription. J Biol Chem 281: 5567–5574. 16407261 10.1074/jbc.M600035200

[27] Vij N, Zeitlin PL (2006) Regulation of the ClC-2 lung epithelial chloride channel by glycosylation of SP1. Am J Respir Cell Mol Biol 34: 754–759. 16456185 10.1165/rcmb.2005-0442OC PMC2644236

[28] Chuang JY, Wu CH, Lai MD, Chang WC, Hung JJ (2009) Overexpression of Sp1 leads to p53-dependent apoptosis in cancer cells. Int J Cancer 125: 2066–2076. 19588484 10.1002/ijc.24563

